# Characterization and immunological effect of outer membrane vesicles from *Pasteurella multocida* on macrophages

**DOI:** 10.1007/s00253-024-13060-2

**Published:** 2024-02-26

**Authors:** Jiaying Sun, Yee Huang, Xuefeng Li, Xiangfei Xu, Xuemei Cui, Fangjiao Hao, Quanan Ji, Chun Chen, Guolian Bao, Yan Liu

**Affiliations:** 1 College of Life Sciences, China Jiliang University, Zhejiang, 310018 Hangzhou China; 2 Institute of Animal Husbandry and Veterinary Science, Zhejiang Academy of Agricultural Sciences, Zhejiang, 310021 Hangzhou China

**Keywords:** *Pasteurella multocida*, Outer membrane vesicles, Proteomics, Inflammatory response

## Abstract

**Abstract:**

*Pasteurella multocida* is an important bacterial pathogen that can cause diseases in both animals and humans. Its elevated morbidity and mortality rates in animals result in substantial economic repercussions within the livestock industry. The prevention of diseases caused by *P. multocida* through immunization is impeded by the absence of a safe and effective vaccine. Outer membrane vesicles (OMVs) secreted from the outer membrane of Gram-negative bacteria are spherical vesicular structures that encompass an array of periplasmic components in conjunction with a diverse assortment of lipids and proteins. These vesicles can induce antibacterial immune responses within the host. *P. multocida* has been shown to produce OMVs. Nonetheless, the precise characteristics and immunomodulatory functions of *P. multocida* OMVs have not been fully elucidated. In this study, OMVs were isolated from *P. multocida* using an ultrafiltration concentration technique, and their morphology, protein constitution, and immunomodulatory properties in RAW264.7 cells were studied. Transmission electron microscopy (TEM) and nanoparticle tracking analysis (NTA) revealed that the OMVs exhibited typical spherical and bilayered lipid vesicular architecture, exhibiting an average diameter of approximately 147.5 nm. The yield of OMVs was 2.6 × 10^11^ particles/mL. Proteomic analysis revealed a high abundance of membrane-associated proteins within *P. multocida* OMVs, with the capability to instigate the host’s immune response. Furthermore, OMVs stimulated the proliferation and cellular uptake of macrophages and triggered the secretion of cytokines, such as TNF-ɑ, IL-1β, IL-6, IL-10, and TGF-β1. Consequently, our results indicated that OMVs from *P. multocida* could directly interact with macrophages and regulate their immune function *in vitro*. These results supported the prospective applicability of *P. multocida* OMVs as a platform in the context of vaccine development.

**Key points:**

*• Preparation and characterization of P. multocida OMVs.*

*• P. multocida OMVs possess a range of antigens and lipoproteins associated with the activation of the immune system.*

*• P. multocida OMVs can activate the proliferation, internalization, and cytokine secretion of macrophages in vitro.*

**Supplementary Information:**

The online version contains supplementary material available at 10.1007/s00253-024-13060-2.

## Introduction

In the 1880s, Louis Pasteur was the first to identify the etiological role of *Pasteurella multocida* (Harper et al. 2006). *P. multocida* is a highly infectious, zoonotic pathogen that can cause numerous diseases of economic importance, including avian cholera, bovine hemorrhagic sepsis, endemic animal pneumonia, and porcine atrophic rhinitis (Boyce and Adler 2006; Roier et al. 2013; Wilkie et al. 2012). Contact with the saliva of animals colonized by *P. multocida* can cause soft tissue infection in humans (Wilson and Ho 2013). Other severe complications, such as pneumonia, meningitis, and sepsis from infection, may also occur in elderly individuals, immunocompromised individuals, and neonates (Piorunek et al. 2023). The importance of *P. multocida* as an agent in human disease has been gradually recognized. The capsule, lipopolysaccharide (LPS), and adhesins are the main virulence determinants and immunogenic structures of *P. multocida* that play a major role in the evasion of the host innate immune barrier and the infection of the host (Boyce and Adler 2000; Harper and Boyce 2017). Vaccine was reported to be an economical and effective antibacterial infection product which could avoid the drawbacks of conventional antibiotic therapy (Wu et al. 2022). Current vaccines against *P. multocida* are mainly inactivated bacteria and live attenuated bacteria. However, these vaccines have been reported to have limited immunogenicity, reactogenicity, and potential reversion to virulence (Mostaan et al. 2021). Therefore, it would be desirable to develop a novel subunit vaccine that is both safe and protective.

Bacterial membrane vesicles (MVs) were first recognized as being generated through the outer membrane budding process in Gram-negative bacteria, thus generating the designation of outer membrane vesicles (OMVs) (Gan et al. 2021). OMVs can enhance bacterial interactions with the environment, promote bacterial pathogenesis, increase bacterial viability, and modulate interactions within the microbiota (Schwechheimer and Kuehn 2015). OMVs exhibit a spherical morphology and consist of bilayered lipid membrane nanostructures, typically measuring between 20 and 250 nm in size (Kulp and Kuehn 2010). OMVs contain numerous bacterial components, including lipids, proteins, pathogen-associated molecular patterns (PAMPs), adhesins, and peptidoglycan (Kaparakis-Liaskos and Ferrero 2015). The coexistence of bacterial antigens and multiple PAMPs with immunostimulatory properties and the nanoscale structure of protein-lipid complexes make OMVs promising vaccine candidates for the prevention and treatment of bacterial infection (Lieberman 2022; van der Pol et al. 2015).

OMVs have been reported to induce antigen presentation to elicit an antigen-specific immune response by delivering both antigens and adjuvants from PAMPs to antigen-presenting cells (APCs) (Li et al. 2020). Macrophages are one of the important APCs in the innate immune system and perform key functions including phagocytosis, migration, the initiation of inflammatory responses, cytokine production, and presentation of antigens to T cells (Di Benedetto et al. 2019; Mosser and Edwards 2008). These cells express a range of innate immune receptors, including scavenger receptors, mannose-binding lectin (MBL), Fcγ receptors, toll-like receptors (TLRs), and C-type lectin-like receptors (CLRs), which are distributed throughout the cell membrane, cytoplasm, and inner membrane chamber (Mosser and Edwards 2008). During bacterial infection, macrophages recognize PAMPs via surface-exposed, vesicular, or cytoplasmic pattern recognition receptors (PRRs). Then, they phagocytose invading pathogens and release inflammatory mediators to facilitate both innate and adaptive immune responses (Weiss and Schaible 2015).

Owing to their immunogenicity and adjuvanticity, bacterial OMVs have garnered increasing attention as potential vaccines. Research has indicated that OMVs have the ability to elicit a defensive immune response in animals (Roier et al. 2013). To investigate the efficacy of OMVs as vaccinal components for preventing *P. multocida* infection, we isolated OMVs from *P. multocida* cultures and explored their morphological traits. Then, we studied the protein composition of the sample using proteomics techniques, which revealed additional information about their contents and potential functions. Finally, we investigated their role in the immune activation of macrophages by analyzing the proliferation, uptake, and cytokine secretion by RAW264.7 macrophages *in vitro*.

## Materials and methods

### Bacterial culture and isolation of OMVs

*P. multocida* (China Veterinary Drug Supervision Institute no. CVCC500) was cultured aerobically in Martin broth (MB) at 37 °C for 18 h, shaken at 200 rpm, replenished with a 1:100 dilution of MB, and allowed to grow for an additional 14 h until the stationary phase was reached. The cultured mixture was centrifuged at 5000 × g for 10 min at 4 °C, and the supernatant was subjected to a second round of centrifugation under the same conditions. The supernatant containing the OMVs was consecutively filtered through 0.45-μm and 0.22-μm filter membranes to remove live bacteria and cell debris. The filtrate was subsequently concentrated using concentrators with a filter membrane of 100 kDa (Millipore, USA). The resulting concentrated filtrate was ultracentrifuged at 100,000 × *g* for 2 h at 4 °C to pellet the OMVs. Finally, the OMV deposit pellet was resuspended in 1×phosphate-buffered saline (PBS), and the protein concentration was assessed using a bicinchoninic acid (BCA) assay (Solarbio, China). The prepared OMVs were stored at −80 °C until use.

### Particle size, concentration, and zeta potential

The particle size–concentration and potential–concentration of the OMVs were measured via NTA (Zetaview, Particle Metrix, Germany). OMVs were diluted with a sterile PBS buffer at a ratio of 1:5000, and the average number of counted particles per frame was approximately 100–120. Then, we selected the NTA measurements from 11 distinct positions for recording and analysis.

The quantity of OMVs per unit (CFU) released by *P. multocida* was assessed by integrating the quantified number of OMVs using NTA with the count of colonies in the original culture obtained through colony counting.

### Transmission electron microscopy

For TEM analysis, OMVs were isolated as previously described, deposited onto a 200-mesh copper grid, and stained with uranyl acetate before they were dried at room temperature. Imaging procedures were carried out using a Tecnai^TM^ G2 Spirit BioTWIN electron microscope operating at 80 kV.

### SDS–PAGE

Protein (50 μg) from the entire-cell lysates and protein (50 μg) from OMVs prepared as previously described underwent analysis with sodium dodecyl sulfate-polyacrylamide gel electrophoresis (SDS–PAGE, 12% resolving gel). The resulting gel was stained with Coomassie Brilliant Blue R-250 dye.

### Proteomic analysis of OMVs

Library construction and qualitative proteome analysis were carried out by Novogene Biotech (Beijing, China). The OMV sample containing 50 μg of protein was used for qualitative proteome analysis. OMV samples were collected and mixed with DB protein solution (8 M urea, 100 mM triethylammonium bicarbonate [TEAB]) and subjected to trypsin digestion at 37 °C for 4 h, followed by overnight digestion with trypsin and CaCl_2_. Subsequently, the digested OMV samples were mixed with formic acid, loaded onto a C18 desalting column, and washed with 0.1% formic acid and 3% acetonitrile washing buffer. This was succeeded by elution using a buffer consisting of 0.1% formic acid and 70% acetonitrile. The eluents were collected and freeze-dried for subsequent LC-MS/MS analysis.

Tryptic peptides were separated using an EASY-nLC^TM^ 1200 UHPLC system (Thermo Fisher, Germany). Peptides eluted from OMV samples were analyzed using a Q ExactiveTM HF-X mass spectrometer (Thermo Fisher, Germany) operating in data-dependent mode with a Nanospray Flex™ (ESI) ion source. The overall scanning range spanned m/z 350 to 1500 with a resolution of 60,000 (at m/z 200). The full scan precursors were selected based on their high-to-low abundance and then fragmented via higher energy collisional dissociation (HCD). The process was carried out with a resolution of 15000 (at m/z 200), and the normalized collision energy was set to 27%. The resulting MS/MS spectra were recorded in the linear ion trap.

The resulting spectra were searched separately against the UniProt *Pasteurella_multocida* database (1632170-Uniprot-*Pasteurella_multocida*.fasta (23309 sequences)) using Proteome Discoverer (PD 2.5, Thermo Fisher). The search was performed with a maximum of two allowed missed cleavages, applying carbamidomethyl as a fixed modification, oxidation of methionine (M) as a dynamic modification, and methionine at the N-terminus as a loss. The precursor ion’s mass tolerance was set at 10 ppm, and for production, it was 0.02 Da. PD2.5 was used to analyze the retrieval results, ensuring that the identified peptide spectrum matches (PSMs) exhibited over 99% credibility. The identified proteins were required to contain at least one unique peptide, and all identified PSMs and proteins were subjected to analysis with a false discovery rate (FDR) of no more than 1.0%. Cell-mPLOC 2.0 was used to predict subcellular localization. To perform Gene Ontology (GO) functional analysis, InterproScan (version 5.22-61.0) was employed and compared with the Pfam database (http://pfam.xfam.org/). The identified proteins were subjected to BLAST searches against the online Kyoto Encyclopedia of Genes and Genomes (KEGG) database (http://www.genome.jp/kegg/) and Clusters of Orthologous Groups (COG) database (http://www.ncbi.nlm.nih.gov/COG/) (BLASTP, evalue ≤ 1e-4), after which the results were compared with the highest score to annotate the protein families and pathways.

### Cell cultures

The RAW264.7 murine macrophage line was obtained from the Chinese Academy of Science Cell Bank and cultured in DMEM (Gibco, USA) supplemented with 10% FBS, 50 U/mL penicillin (Gibco, USA), and 50 μg/mL streptomycin (Gibco, USA). The cells were maintained at 37 °C and 5% CO_2_, and the medium was changed every 48 h.

### Cell proliferation assay

The impact of OMVs on cell growth was evaluated using CCK-8 assay (APExBIO, USA). RAW264.7 macrophages were initially seeded into 96-well plates at a density of 5.0 × 10^3^ per well and then incubated in a humid environment at 37 °C with 5% CO_2_ until adherence to the well surface occurred. After this initial phase, the cells were stimulated with 100 μL of fresh medium containing various concentrations of OMVs (62.5 μg/mL, 12.5 μg/mL, 2.5 μg/mL, 0.5 μg/mL, and 0.1 μg/mL), in addition to a positive control of LPS at 10 μg/mL. The negative control consisted of medium alone (referred to as BC). The cell cultures were then incubated for 48 h at 37 °C under 5% CO_2_. Each concentration was tested in 6 wells. Following incubation, the infected plates were maintained at 37 °C with a 5% CO_2_ environment for an additional 0.5–2 hours, after which 10 μL of CCK-8 reagent was added to each well. Finally, the growth and proliferation of the cells were determined by measuring the optical density (OD) at 450 nm.

### Cellular uptake and colocalization

The pelleted OMVs were fluorescently labeled with DiD (DiIC18(5), Yeasen, China) for 30 min at 37 °C for internalization by macrophage RAW264.7 cells. RAW264.7 cells were plated at a density of 5 × 10^5^ cells per well on 8-well slides (Ibidi, USA) and subsequently incubated at 37 °C in a 5% CO_2_ environment for 24 h. Then, 100 μL of labeled OMVs was added at a concentration of 5 μg/mL of protein into cells in 200 μL of medium, and the slides were incubated for 1 h, 2 h, and 4 h under 5% CO_2_ at 37 °C. The supernatant was discarded, and the cells were subjected to three washes with PBS and stained with LysoTracker Green DND-26 (Yeasen, China) to label the lysosomes. After 1 h of incubation, Hoechst 33342 (APExBIO, USA) was added for nuclear staining. After rinsing with PBS, the cells were fixed using 4% paraformaldehyde. Subsequently, the images were analyzed utilizing a confocal laser scanning microscope (Leica TCS-SP5).

To quantitatively and dynamically evaluate cellular uptake, RAW264.7 macrophages were cultured at a density of 5 × 10^6^ cells per well in 6-well plates at 37 °C in 5% CO_2_ for 24 h. The cells were then exposed to varying concentrations of DiD-labeled OMVs (0.2 μg/mL, 1 μg/mL, and 5 μg/mL) and subsequently incubated at 37 °C in 5% CO_2_ for 1 h, 2 h, and 4 h. Following this, the cells were treated with trypsin and assessed using flow cytometry to determine the uptake of OMVs.

### Cell cytokine secretion

Macrophages can stimulate immune reactions by secreting a diverse array of immune-enhancing cytokines. To investigate whether *P. multocida* OMVs can induce inflammatory responses in macrophages in an extracellular environment, the concentrations of cytokines released from macrophage RAW264.7 cells were quantified. RAW264.7 cells were plated in 24-well plates at a density of 4 × 10^5^ cells per well and cocultured with varying concentrations of OMVs (62.5 μg/mL, 12.5 μg/mL, 2.5 μg/mL, and 0.5 μg/mL), as well as LPS (10 μg/mL) and medium alone as positive and negative controls, respectively. Six replicates were conducted for each concentration. The cells were then incubated at 37 °C in a 5% CO_2_ environment for 24 h. Following this, TNF-ɑ, IL-1β, IL-10, TGF-β1, and IL-6 were quantitatively assessed using a mouse ELISA Kit (Lianke Bio, China) after the culture supernatant was collected. The detection process strictly followed the manufacturer’s instructions.

### Statistical analysis

The data were analyzed and visualized using GraphPad Prism software (version 9.5) and are presented as the mean ± standard error (SE) deviation. The “*n*” value represents the number of independent experiments. Each measurement was performed three times and carried out independently in separate experiments (*n*). Statistically significant results were analyzed using a *t*-test, and a one-way analysis of variance (ANOVA) was performed with Tukey’s multiple comparison test to assess the differences.

## Results

### Characterization of OMVs from *P. multocida*

OMVs were isolated from late logarithmic-phase *P. multocida* cultures cultivated in MB medium by the ultrafiltration concentration technique, and then the morphology and yield of the derived OMVs were studied.

**Fig. 1:**
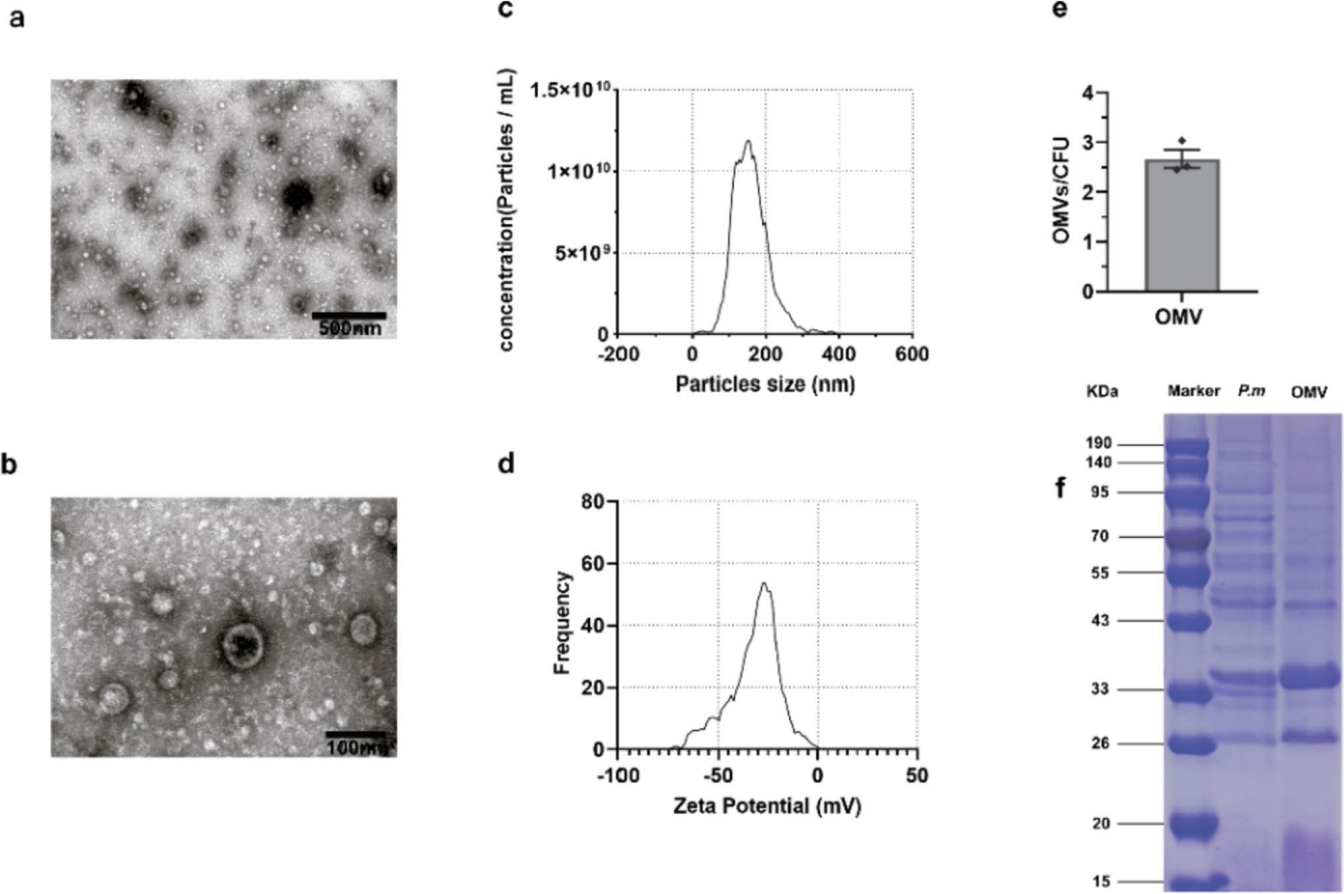
Physical characteristics of OMVs derived from *P. multocida*. Transmission electron micrographs of OMVs (**a**, **b**). NTA results of average OMV hydrodynamic diameter and OMV size distribution (**c**). The zeta potential of the OMVs was measured by NTA (**d**). The number of OMV particles ascertained by NTA was compared to the number of CFU in bacterial cultures to establish the OMVs number of released per CFU (**e**). SDS–PAGE analysis was conducted on whole-cell lysates and OMVs, with lane 1 showing a 190-kDa protein ladder, lane 2 representing *P. multocida* whole-cell lysates, and lane 3 displaying *P. multocida* OMVs (**f**)

The size, shape, and structure of the OMVs were visualized using TEM, which is a technique that has been frequently used for the determination of the size, shape, and cellular activities of various biological samples in previous studies (Agrawal et al. 2020). TEM result showed that the purified OMVs exhibited an irregular spherical, bilayer lipid vesicle architecture of variable size, with diameters spanning from 20 to 300 nm (Fig. 1a, b). A study has suggested that OMVs formed through blistering of the outer membrane should consist of a single bilayer membrane. However, our TEM images revealed a bilayer membrane structure for the OMVs. This observation raises the possibility that the OMVs prepared using our current method may indeed represent outer-inner membrane vesicles (OIMVs) generated by explosive cell lysis triggered by phage-derived intracellular lysins (Toyofuku et al. 2019).

NTA was chosen for the monitoring and analysis of the size distribution, concentration, and zeta potential of OMVs in this study due to its superior detection limit at low concentrations compared to that of dynamic light scattering (DLS) (Mourdikoudis et al. 2018). Analysis via NTA showed an average diameter of 147.5 nm for OMVs (Fig. 1c). OMVs measuring approximately 10–50 nm in diameter constitute a small fraction of the population, while larger vesicles with diameters of 100–200 nm make up the majority of the population (Fig. 1c). These results support our previous research on the morphological characterization of OMVs via TEM. The concentration of OMVs was 2.6×10^11^ particles/mL (Fig. 1c), and the zeta potential was −28.86 mV (Fig. 1d). According to the NTA analysis and colony counting from the original liquid culture, an estimated 2.7 OMVs were released per bacterium (Fig. 1e). The data are the average of three independent experiments.

### Proteomic analysis of *P. multocida* OMVs

*P. multocida* OMVs and whole-cell lysates were analyzed using SDS–PAGE. The results revealed the abundance of *P. multocida* proteins in the OMVs. Notably, the number of bands observed in the OMVs was lower compared to whole-cell lysates. However, all protein bands identified in the OMVs corresponded to those in the whole-cell lysate (Fig. 1f). These results demonstrated that the produced OMVs were free from bacterial contamination and contained a diverse array of immunogenic proteins.

Additional information for determining the contents and potential functions of *P. multocida* OMVs was obtained via proteomic analysis. The sequencing of 1500 peptide segments that mapped to 429 proteins of *P. multocida* OMVs was performed via LC–MS/MS. The major proteins identified according to the PSM data are listed in Table 1. Most of the proteins, including filamentous hemagglutinin (FHA), neuraminidase, pilus assembly proteins, pertactin (PRN), structural proteins, binding proteins, and transport proteins, were associated with virulence. In addition, prion proteins, which transport sugars, amino acids, ions, and transmembrane channel proteins, were also found.

**Table 1:** List the important *P. multocida* OMV proteins identified in order of decreasing PSMs

Accession number	Protein name	Molecular mass (kDa)	Gene	Subcellular localization
I2BGA6	Outer membrane protein A	38	ompA	Outer membrane
A0A2J9QJ69	Outer membrane protein A	38	ompA	Outer membrane
V4NAK3	Peptide ABC transporter substrate-binding protein	61.6	P1062_0205060	Periplasm
A0A126QGC9	Tol-Pal system protein TolB	45.9	tolB	Periplasm
A5H9S0	Lipoprotein E	37.5	plpE	--
A0A8E2A638	Sugar ABC transporter substrate-binding protein	33.8	A0R67_09330	Periplasm
A0A191VYV1	Outer membrane protein assembly factor BamA	87.7	bamA	Outer membrane
A0A379BDM0	Hemolysin activation/secretion protein-1	63.5	lspB1_1	Periplasm
A0MCG2	Outer membrane protein	37.2	ompH	Outer membrane
A0A1E3XLL0	Membrane-bound lytic murein transglycosylase C	40.2	mltC	Periplasm
V4PX95	Sialidase	93.3	P1062_0207165	Extracell
A0A1E3XJI9	TolC family protein	50.6	BGK37_07070	Outer membrane
A0A379BCW7	Filamentous hemagglutinin protein	234.5	pfhB1_1	Extracell
Q9CLZ8	Outer membrane protein assembly factor BamC	37.4	bamC	Outer membrane
A0A379BCX4	Protein PfhB2	49.2	pfhB2_2	--
A0A291ID33	Sialidase protein (Fragment)	32.2	nanH	Extracell
A0A8E2A529	Iron ABC transporter substrate-binding protein	35.6	A0R67_08690	--
A0A379BCZ3	Filamentous hemagglutinin protein	81	pfhB1_2	--
A0A1E3XJ59	MipA/OmpV family protein	28.6	BGK37_06210	Outer membrane
A0A2J9QJB6	Pilus assembly protein	67.6	A6J89_002300	--
Q9CLL1	LPS-assembly lipoprotein LptE	18.9	lptE	--
A0A379B9M3	Porin, opacity type	21.7	NCTC10722_00237	Outer membrane
A0A2J9QM57	OMP_b-brl domain-containing protein	26.4	A6J89_006250	--
J5MYK7	Lipoprotein	30.2	AAUPMB_17430	Inner membrane
Q9CNT8	TonB-dependent hemoglobin/transferrin/lactoferrin family receptor	113.3	PM0337	Outer membrane
A1Z0J3	OmpW	21.9	--	Cell outer membrane

**Fig. 2:**
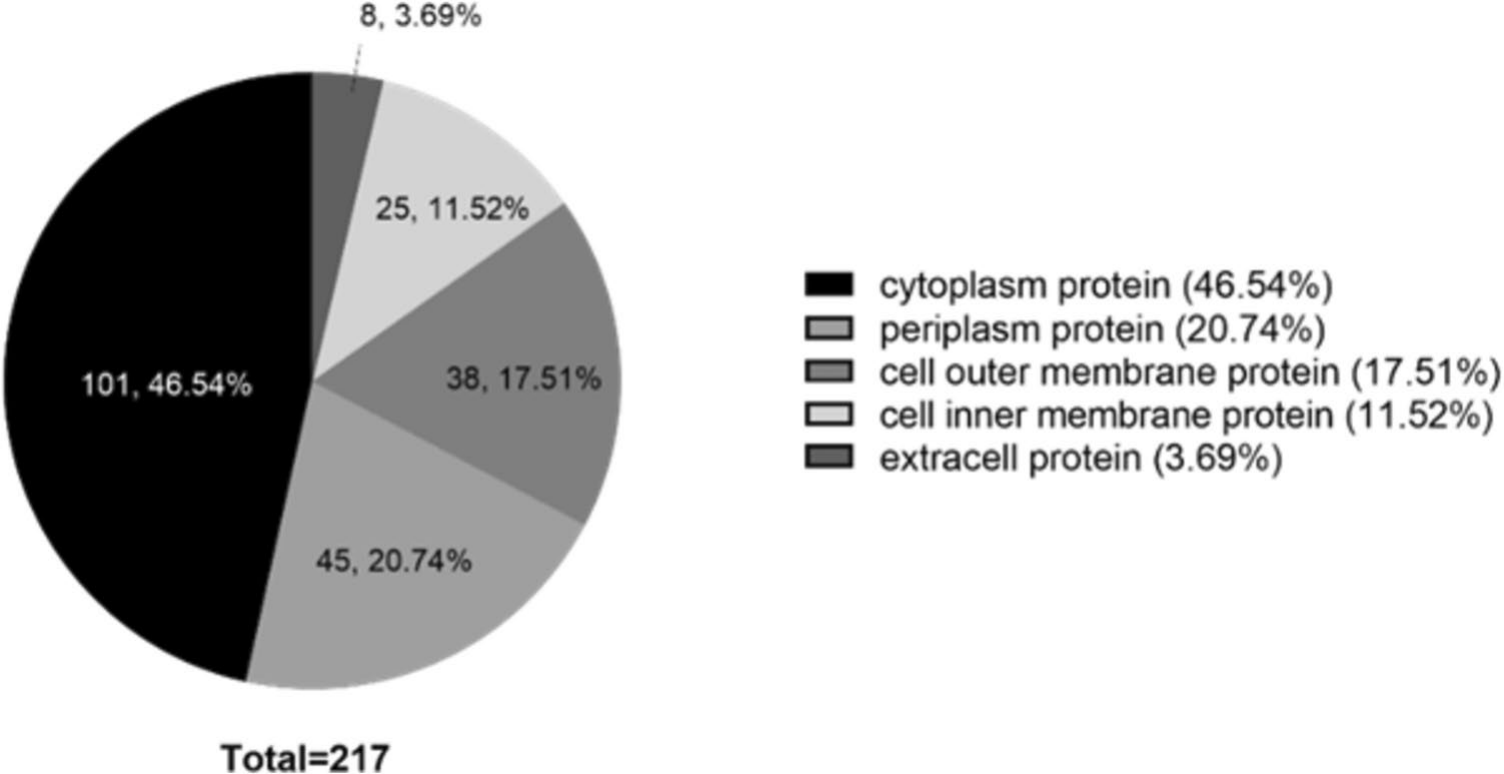
Based on proteomic analysis, the subcellular localization of various proteins within or on the surface of *P. multocida* OMVs was predicted

Based on the analysis of subcellular localization, proteins within OMVs originated from diverse subcellular compartments within the bacterium. Notably, the majority of proteins (46.54%) were classified as cytoplasmic proteins, followed by periplasmic proteins (20.74%) and outer membrane proteins (17.51%) (Fig. 2).

**Fig. 3:**
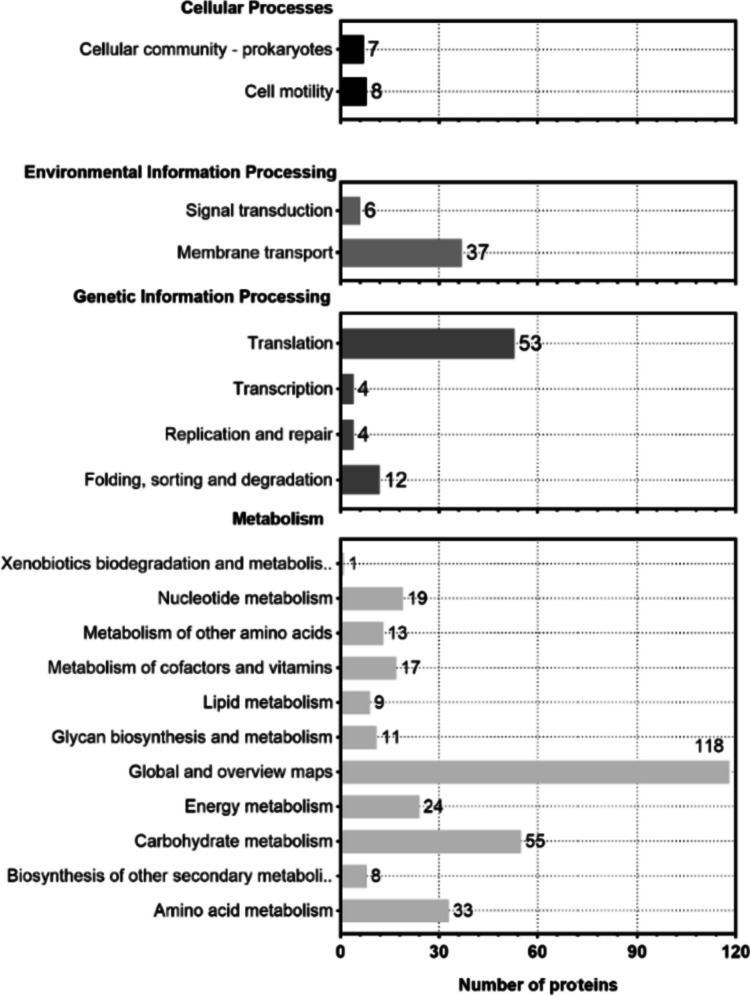
KEGG pathway annotation results of *P. multocida* OMV proteins, which allowed for the analysis of protein pathways

**Fig. 4:**
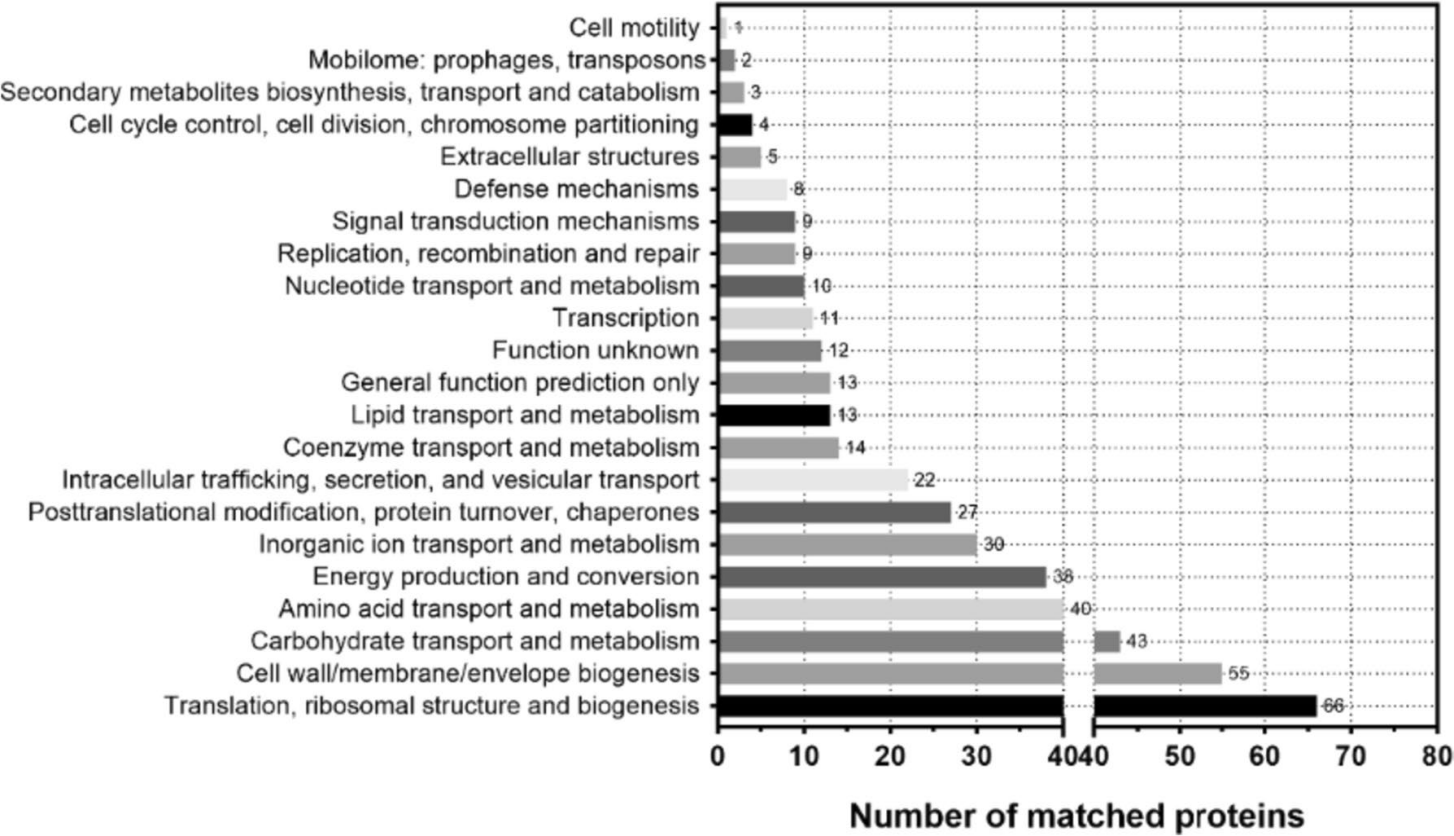
COG functional classification results of the proteins in *P. multocida* OMVs

**Fig. 5:**
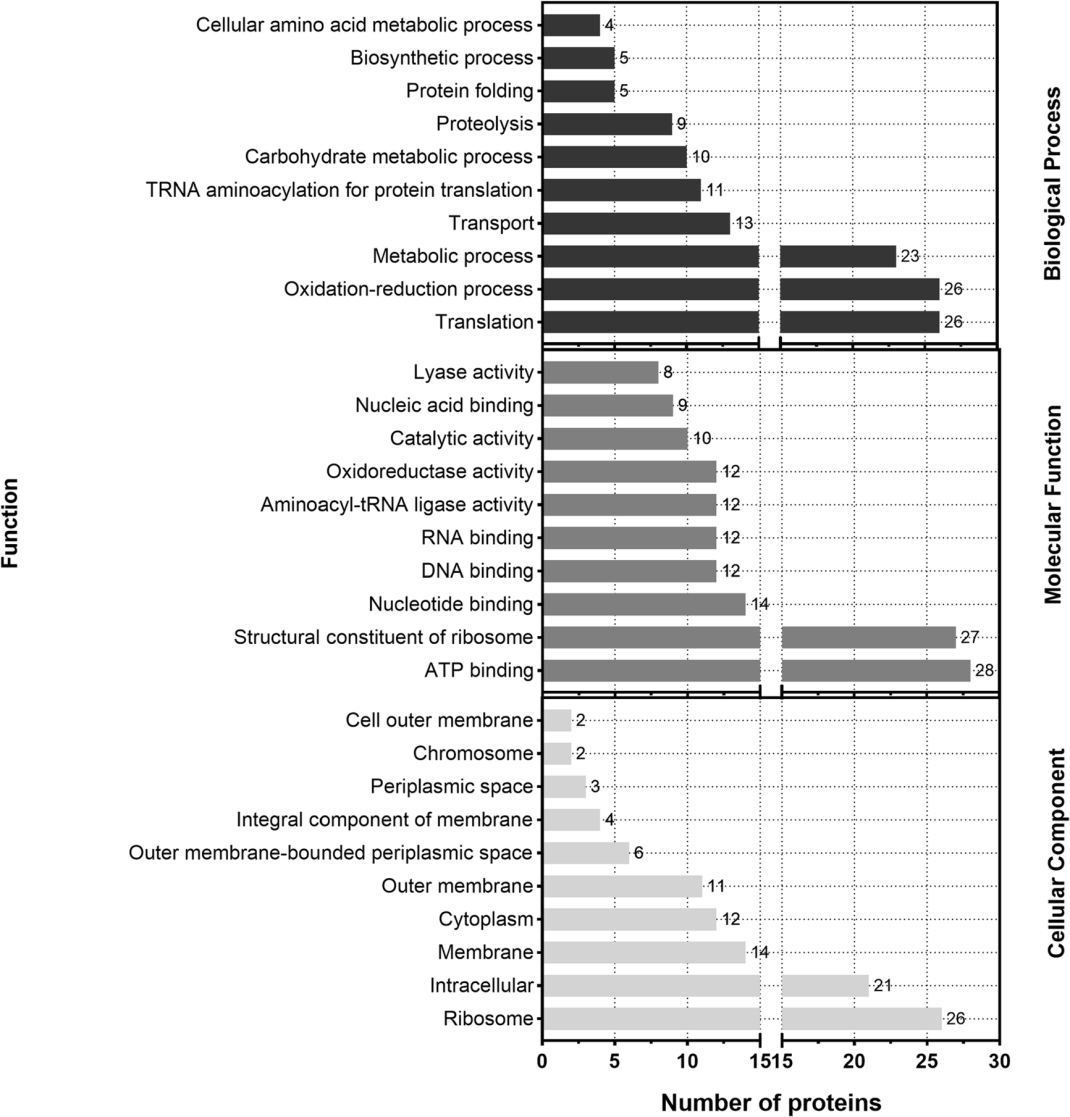
OMV proteins from *P. multocida* classified according to their functions

To identify the involved metabolic and signaling pathways, KEGG pathway analysis was conducted on the OMV proteins. Among the proteins identified in our study, 3.4% are involved in cellular processes, 9.8% are involved in environmental information processing, and 30.5% are involved in genetic information processing and contribute to metabolism. The largest group of proteins is involved in metabolic pathways, which could be attributed to the biogenetic mechanism of OMVs (Fig. 3). The data acquired indicate associations between the majority of vesicle proteins identified in COG analysis and diverse functions of *P. multocida* OMVs. Specifically, 66 proteins are linked to translation, ribosomal structure, and biogenesis, while an additional 55 proteins are associated with the cell wall, membrane, and envelope biogenesis. Moreover, other proteins predominantly play roles in carbohydrate and amino acid transport and metabolism, as well as inorganic ion transport and metabolism. Additionally, these proteins are involved in intracellular transport, secretion, vesicle transport, and defense mechanisms (Fig. 4). Subsequently, we conducted GO enrichment analysis of these proteins. The molecular function terms of the identified proteins encompassed various categories, including biological processes linked to OMV proteins enriched in amino acid, lipid, and carbohydrate transport, as well as oxidation–reduction, metabolism, and translation processes. In terms of cellular components, OMV proteins were enriched in ribosomes, cell membranes, outer membranes, the cytoplasm, and the periplasm. For molecular functions, OMV proteins were enriched in nucleotide and ribonucleotide binding, as was ribosome binding (Fig. 5).

### OMVs stimulate the proliferation of RAW264.7 cells

**Fig. 6:**
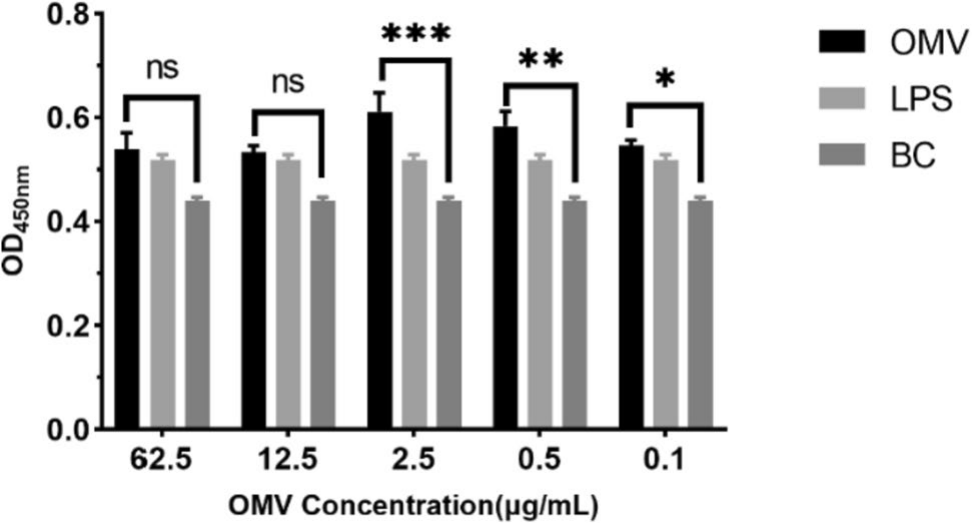
RAW264.7 macrophages stimulated with OMVs from *P. multocida in vitro* (mean ± SE; *n* = 6). LPS served as the positive control, while BC was utilized as the negative control. (*p < 0.05; **p < 0.01; ***p < 0.001; ns: not significant)

In this study, mouse RAW264.7 cells were utilized as the macrophage model due to their established suitability for mimicking macrophage behavior including pinocytosis and phagocytosis (Kong et al. 2019). The proliferation of RAW264.7 macrophages was assessed through CCK-8 assay following exposure to varying concentrations of OMVs. As shown in Fig. 6, RAW264.7 macrophages were stimulated with OMVs ranging from 0.1 to 62.5 μg/mL (*n* = 6). LPS served as the positive control, while BC was utilized as the negative control. The results indicated that OMVs ranging from 0.1 to 2.5 μg/mL could significantly increase the proliferation of RAW264.7 cells compared with BC (negative control). OMVs ranging from 12.5 to 62.5 μg/mL also showed a promotion effect, but was not significant compared with BC.

### OMVs were successfully taken up by RAW264.7 cells

To investigate whether OMVs derived from *P. multocida* can be efficiently phagocytosed by APCs, confocal laser scanning microscopy and flow cytometry were employed to evaluate the interaction between macrophages and DiD-labeled OMVs.

**Fig. 7:**
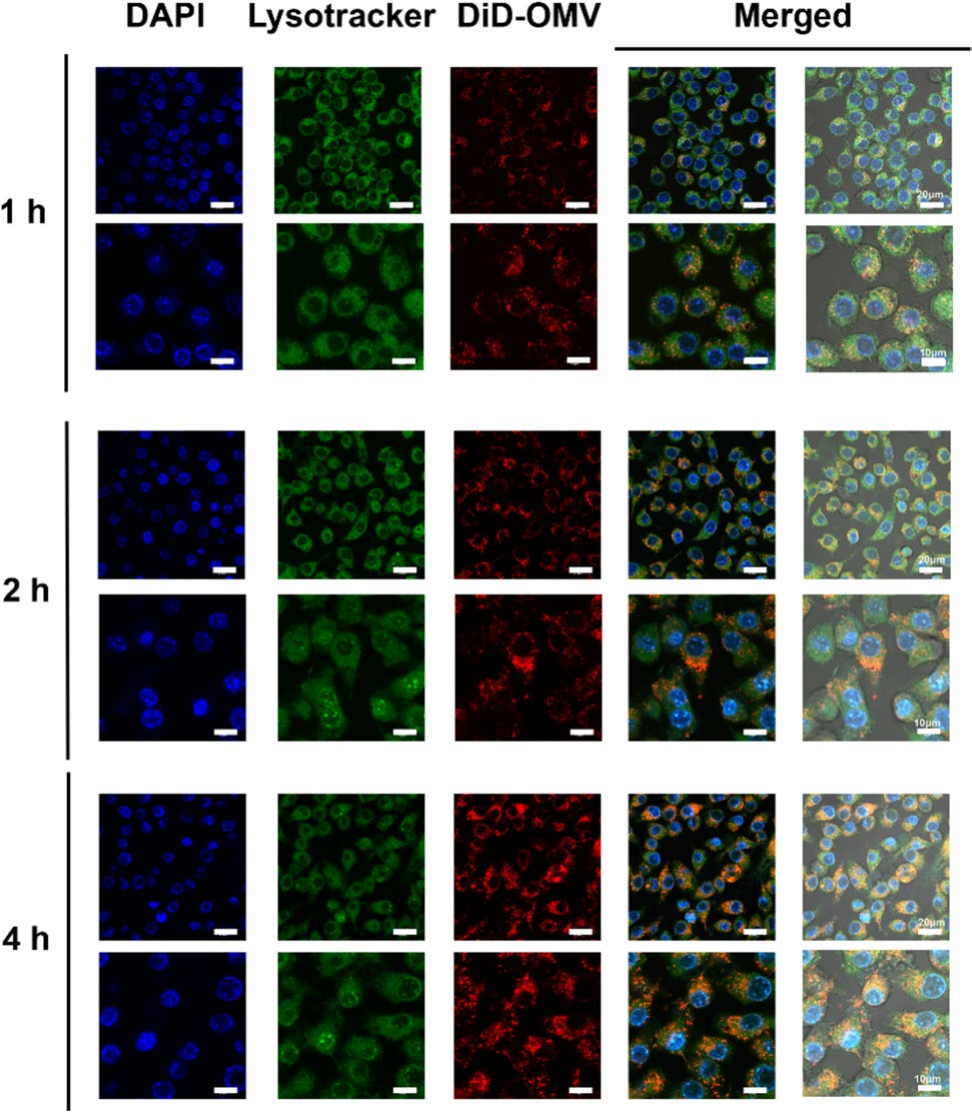
Confocal laser scanning microscopy (CLSM) of DiD (red) labeled OMVs from *P. multocida* uptake by RAW264.7 macrophages at 1 h (up row: 20 μm; down row: 10 μm), 2 h (up row: 20 μm; down row: 10 μm), and 4 h (up row: 20 μm; down row: 10 μm). LysoTracker Green DND-26 (green) was used for lysosome labeling, and Hoechst 33342 (blue) was utilized for nuclear staining. Representative images from three independent experiments are presented

Confocal laser scanning microscopy images clearly demonstrated the clearly illustrating the successful uptake of *P. multocida* OMVs by macrophages. After 1 h of incubation, a limited number of DiD-labeled OMVs were observed within the cells, with minimal intracellular localization. However, over the subsequent 2- and 4-h periods, there was a gradual increase in vesicular internalization within the macrophages (Fig. 7). Notably, macrophages exhibited notably high phagocytosis efficiency, with nearly all the cells demonstrating internalization of the vesicles within 4 h of incubation.

**Fig. 8:**
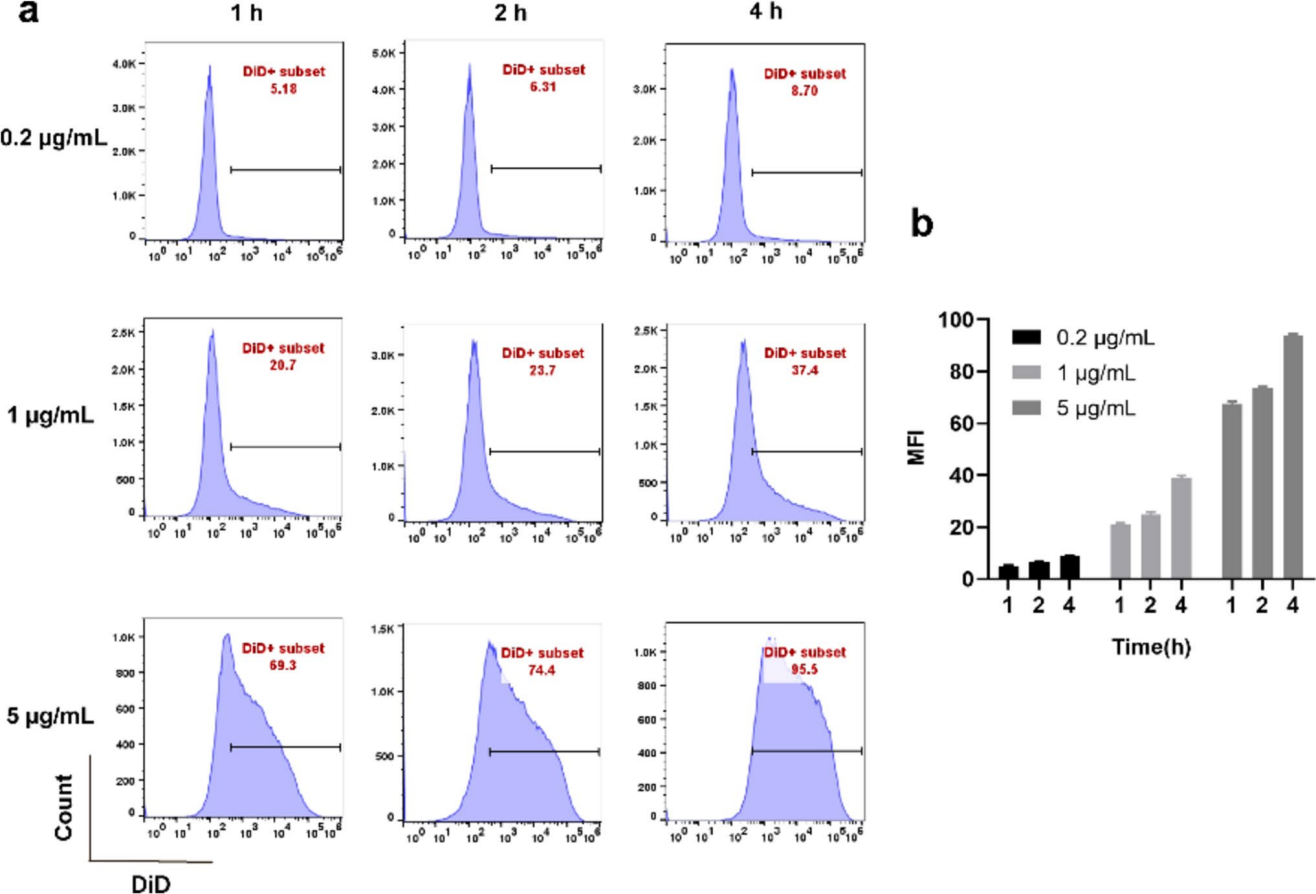
Uptake of DiD-labeled OMVs (0.2 μg/mL, 1 μg/mL, and 5 μg/mL) from *P. multocida* into RAW264.7 macrophages at 1 h, 2 h, and 4 h was quantified via flow cytometry (**a**). The quantification of the flow cytometry data (**b**). Average number of independent experiments (*n* = 3)

A flow cytometry assay was applied to quantitatively analyze the uptake of DiD-labeled OMVs by RAW264.7 macrophages. Figure 8a shows representative data from three repetitions of different treatments, and Fig. 8b shows the quantitative data. The results revealed rapid interactions after a 1-h incubation period. The phagocytosis rates of the low-, medium-, and high-concentration groups were 5.18%, 20.7%, and 69.3%, respectively. By the 2nd hour, the phagocytosis rates modestly increased to 6.31%, 23.7%, and 74.4%, respectively. After 4 h of incubation, the percentage of cells increased to 8.7%, 37.4%, and 95.5% respectively. The nearly complete uptake of OMVs within the high-concentration group was consistent with previous observations highlighting the substantial phagocytosis efficiency of *P. multocida* OMVs by RAW264.7 macrophages.

### OMVs induced inflammatory cytokine release from RAW264.7 cells

**Fig. 9:**
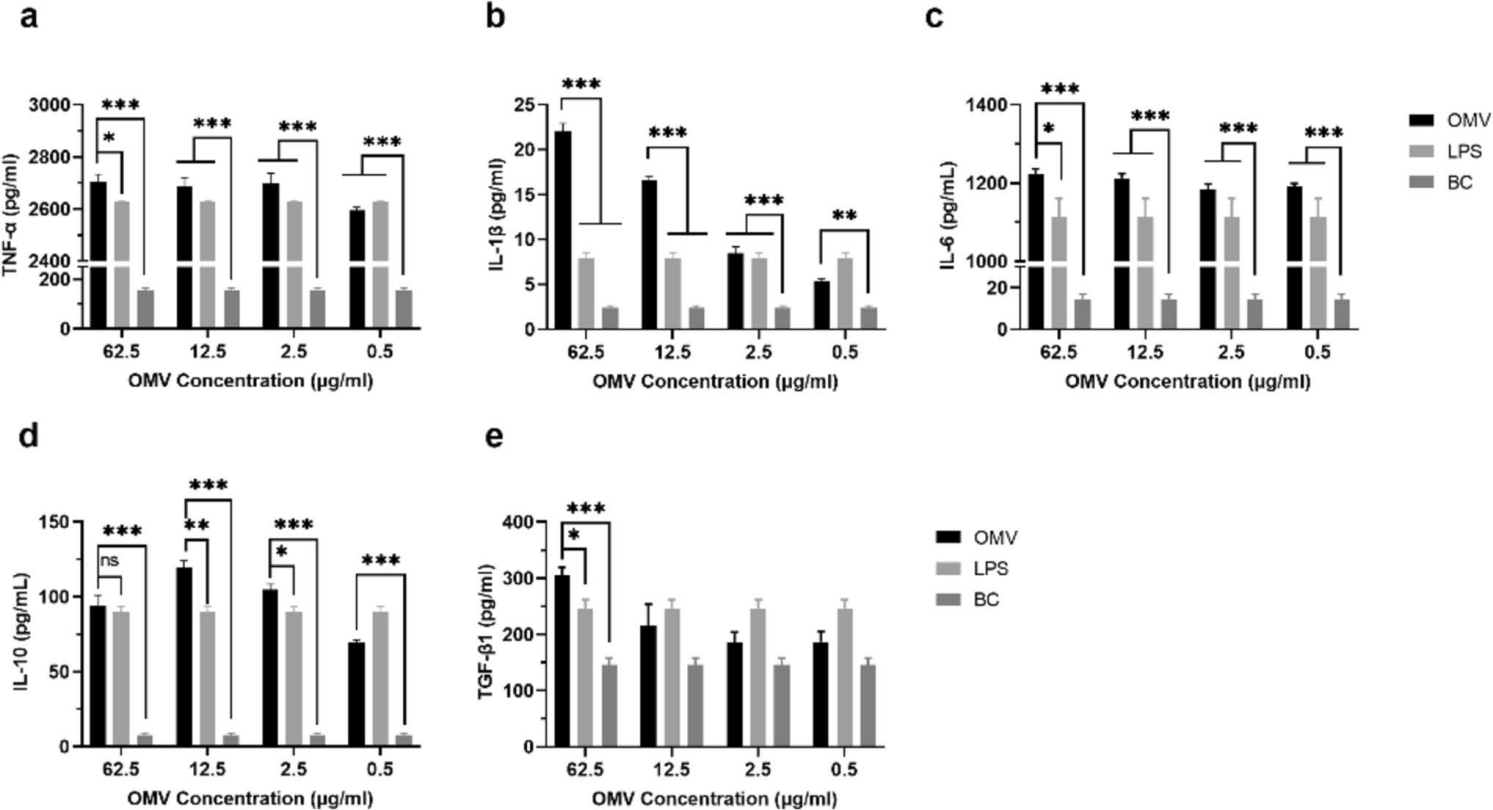
Immunostimulatory cytokines released by RAW264.7 cells incubated with OMVs from *P. multocida*. The levels of TNF-ɑ (**a**), IL-1β (**b**), IL-6 (**c**), IL-10 (**d**), and TGF-β1 (**e**) were quantified using ELISA. (*p< 0.05, **p < 0.01, ***p < 0.001; mean ± SE; *n* = 5)

The production of cytokines by RAW264.7 cells stimulated with OMVs was assessed using ELISA to investigate the immune activation effects of OMVs on macrophages *in vitro* (Fig. 9). Our results demonstrated that significant increases in the production of TNF-ɑ, IL-1β, IL-10, and IL-6 in cells were stimulated with 2.5, 12.5, and 62.5 μg/mL OMV protein, compared to those in the negative control (DMEM) (Fig. 9a–d). Notably, TNF-ɑ and IL-6 by *P. multocida* OMVs suggest the potential concurrent induction of significant Th1 and Th2 immune responses. Moreover, higher levels of the cytokine TGF-β1 were observed at a higher OMV protein concentration (62.5 μg/mL) (Fig. 9e). These findings highlighted a dose-dependent correlation between the secretion of these five cytokines by RAW264.7 cells in response to OMVs and the capacity of *P. multocida* OMVs to stimulate cytokine secretion. Importantly, OMVs triggered markedly higher levels of TNF-ɑ secretion compared to those of the other four cytokines.

## Discussion

OMVs are primarily enriched in PAMPs, including LPS, phospholipids, nucleic materials, outer membrane–associated proteins, and periplasmic molecules. These substances have the potential to potentially interact with immune cells and initiate a protective immune response (Gan et al. 2021; Tiku and Tan 2021). OMVs have recently shown promising prospects in biomedical applications, for example, as vaccines, adjuvants, drug delivery vehicles, and antibacterial adhesion agents (Acevedo et al. 2014). By investigating the biophysical characteristics, protein composition, and immunomodulatory effect of *P. multocida* OMVs on immune cells, we aimed to establish a foundation for *P. multocida* OMV-based vaccine development.

We initially extracted *P. multocida* OMVs using the ultrafiltration enrichment method. Subsequent analysis via TEM and NTA identified the characteristic double-membrane irregular spherical nanostructure of the *P. multocida* OMVs. The average diameter was 147.5 nm, which was marginally smaller than that previously reported, suggesting potential variations arising from distinct preparatory conditions or characterization methodologies (Fernández-Rojas et al. 2014).

Proteomic analysis of the full spectrum proteins of *P. multocida* OMVs aimed to elucidate their specific protein composition and functionality, with the aim of exploring their potential applicability.

Subcellular localization analysis indicated that, in addition to outer membrane proteins, *P. multocida* OMVs prepared using the ultrafiltration concentration method also contain cytoplasmic, periplasmic, and intracellular proteins. Previously, such observations were attributed to sample contamination with inner membrane proteins and cytoplasmic proteins. However, recent studies have revealed that distinct pathways for MV production result in diverse vesicle subtypes. The explosive cell lysis pathway involves the formation of vesicles through fragment coiling and self-fracturing; these vesicles include proteins from the outer membrane, periplasm, inner membrane, cytoplasm DNA, and peptidoglycan (Toyofuku et al. 2019; Turnbull et al. 2016).

Among the proteins involved in our comprehensive functional analysis, a significant proportion was associated with translation, ribosomal structure, and biogenesis processes, which may have a correlation with the pathway of OMV formation. The subsequent prominent category consisted of proteins involved in cell wall/membrane/envelope biogenesis processes. Studies have shown that the bacterial outer membrane contains transmembrane proteins and lipoproteins, which can act as virulence factors and enzymes, activating the innate immune system and playing a crucial role in the immune response following host infection (Smithers et al. 2021; Verma et al. 2013).

We discovered several immunogenic proteins within the functional category of cell wall/membrane/coating biosynthesis (Supplementary Table S1). Among these proteins, OmpA (A0A2J9QJ69, I2BGA6) exhibited the highest number of PSMs, OmpA originates from the outer membrane, and its molecular weight is 38 kDa. According to the KEGG database, these proteins are classified as opaque proteins or related surface antigens. Moreover, OmpA is a versatile protein with adhesin activity that mediates the formation of bacterial biofilms, stimulates the production of proinflammatory cytokines, and participates in the adhesion and invasion of host cells by bacteria (Dabo et al. 2003; E-Kobon et al. 2017; Katoch et al. 2014; McClean 2012; Yang et al. 2023). Additionally, OmpH (A0MCG2) is a 37.2-kDa molecule originating from the outer membrane. KEGG describes it as a porin outer membrane protein. However, studies have shown that OmpH is a virulence protein that facilitates the diffusion of diverse molecules and possesses specific and cross-reactive epitopes that are abundantly expressed on the bacterial surface. In animal models, OmpH can induce protective immunity against *P. multocida* (Tan et al. 2010). Furthermore, BamA (A0A191VYV1) is also an immunogenic protein associated with *P. multocida* antigens, with a molecular weight of 87.7 kDa and originating from the outer membrane. BamA belongs to the outer membrane protein insertase family and is functionally classified as a bacterial surface antigen according to its COG database.

In other COG functional categories, we also identified filamentous hemagglutinin protein (A0A379BCW7) and sialidase (V4PX95). Filamentous hemagglutinin, as classified in the GO system, is a protein associated with pathogenic mechanisms*.* It serves as a potential virulence factor, facilitating bacterial adhesion to host cells (Tatum et al. 2005). Sialidases, on the other hand, can be generated by various respiratory mucosal pathogens and promote the adhesion and colonization of bacteria (Xu et al. 2009). Notably, both proteins are extracellular, possibly because explosive cell lysis may occur during bacterial culture. After ultracentrifugation, extracellular proteins appear in the OMVs. Furthermore, lipoprotein E (A5H9S0) has also been identified. Lipoprotein E plays a critical role as an immunogenic membrane protein with variable serotype coverage, immunogenicity, antigenicity, and accessible antibodies and is responsible for facilitating complement-mediated killing (Li et al. 2022). Our findings show that *P*. *multocida* OMVs are abundant in immunogenic proteins. We also identified the peptide ABC transporter substrate-binding protein (V4NAK3), which is derived from a periplasmic protein and involved in the quorum-sensing process of *P. multocida* by KEGG. TonB-dependent hemoglobin (Q9CNT8) plays a key role in iron uptake as a virulence factor for bacteria that ensures survival within the host (Krewulak and Vogel 2008). TolC acts as an outer membrane transporter, participating in the type I secretion of macromolecular proteins and the removal of smaller, toxic compounds (Buchanan 2001).

Taken together with previously reported results, KEGG pathway analysis, COG analysis, and GO enrichment analysis indicated that the proteins contained within *P. multocida* OMVs likely participate in various processes. In addition to activating and regulating the host immune response, these proteins may also modulate the biogenesis of OMVs. A study proposed a new mechanism of OMVs formation in which the peptidoglycan layer of a bacterial cell is weakened by autolysin, allowing the inner membrane to protrude into the periplasm, and the vesicles are eventually pinched away from the cell surface along with the surrounding outer membrane (Toyofuku et al. 2019). Proteins involved in cell wall/membrane biogenesis, including OmpA, OmpW, and MipA (Supplementary Table S1), may play a role in this process (Moon et al. 2012; Lee et al. 2007). Furthermore, the protein cargo of *P. multocida* OMVs is likely to play a critical role in promoting bacterial survival and facilitating communication with other bacteria. However, additional experiments are necessary to validate these functions.

In summary, our data revealed abundant antigenic proteins associated with virulence and infection mechanisms in isolated *P. multocida* OMVs. These proteins are crucial for bacterial invasion into the host, suggesting that OMVs could be promising candidates for vaccine development against *P. multocida*. However, the presence of proteins with unknown functions in *P. multocida* OMVs highlights the need for further exploration and investigation.

Macrophages play a crucial role in the body’s initial response to pathogen invasion and perform a range of functions such as phagocytosis, migration, cytokine secretion, antigen presentation, and initiation of inflammatory responses (Pidwill et al. 2020; Weiss and Schaible 2015). These functions collectively contribute to the body’s defense against pathogens. We cultured *P. multocida* OMVs and RAW264.7 macrophages to investigate the potential of *P. multocida* OMVs to modulate cellular responses in an *in vitro* setting.

The internalization and eradication of pathogens by macrophages are key parts of the innate immune response and contribute to antigen presentation and the development of acquired immunity. Phagocytosis is an essential process in macrophages (Chen et al. 2023). Through the insights gained from confocal microscopy and flow cytometry, it was observed that *P. multocida* OMVs were swiftly internalized by RAW264.7 macrophages and were distributed randomly within the lysosomes of the macrophages. This finding underscores the potential for the relevant antigens present on the surface of *P. multocida* OMVs to be presented, facilitating their recognition and uptake by macrophages, thereby delivering antigen information.

Macrophages, as professional APCs, recognize bacteria through PRRs and subsequently bind bacteria to initiate phagocytosis (Weiss and Schaible 2015). This process allows them to phagocytose and digest pathogens, presenting antigens to T cells through major histocompatibility complex II (MHC-II), establishing a connection between the innate and adaptive immune systems. This sequential process plays a pivotal role in the initiation of adaptive immune responses (Dale et al. 2008). Furthermore, this process triggers intracellular reactions that lead to macrophage proliferation and enhanced immune function (Ren et al. 2017).

Our quantitative analysis of macrophage proliferation induced by *P. multocida* OMVs using the CCK-8 method yielded evidence indicating that macrophage RAW264.7 cells underwent proliferation upon acquiring antigen information from *P. multocida* OMVs. This discovery supports the notion that *P. multocida* OMVs contribute to macrophage proliferation and activation in an *in vitro* setting. Additionally, upon sensing pathogenic signals, macrophages release cytokines to facilitate both innate and adaptive immune responses (Brown 2006; Weiss and Schaible 2015). Macrophages can be categorized into M1 and M2 types, each of which is involved in distinct immune responses. M1 macrophages are associated with type 1 immune responses and play a role in killing intracellular pathogens, characterized by heightened synthesis of proinflammatory cytokines such as IL-6, TNF-ɑ, and IL-1β. In contrast, M2-type macrophages are involved in immune regulation and promote the type 2 immune response by producing inflammatory chemokines such as TGF-β1 and IL-10 (Zhang and Wang 2014; Shapouri-Moghaddam et al. 2018). The findings revealed that OMVs derived from *P. multocida* were capable of promoting cytokine secretion in RAW264.7 cells. Notably, compared with the other four cytokines, OMVs significantly increased the secretion of TNF-ɑ in RAW264.7 cells. TNF-ɑ, produced by effector T cells or innate immune cells, has been shown to activate both CD4^+^ and CD8^+^ T cells, enhance T-cell proliferation, and increase cytokine production (Mehta et al. 2018). Additionally, the levels of IL-6 were higher than those of the other three cytokines except TNF-ɑ, potentially due to the regulatory effect of TNF-ɑ on stimulating IL-6 secretion. IL-6 serves as a link between the immune responses of T cells and B cells, contributes to the specific differentiation of naive CD4 T cells, and prompts the differentiation of CD8 T cells into cytotoxic T cells. This linkage effectively bridges the innate and adaptive immune responses in the host following *P. multocida* infection, thereby playing a central role in anti-infection defense. Furthermore, IL-6 stimulates lymphocytes and facilitates the production of antibodies, which are essential for eliminating *P. multocida* within the host (Tanaka et al. 2014; Tanaka et al. 2016). Furthermore, the results showed that *P. multocida* OMVs also stimulated the secretion of Th1-type polarized cytokines (IL-1β) and Th2-type polarized cytokines (IL-10 and TGF-β1) in macrophages. IL-1β promotes B-cell proliferation and, in conjunction with antigens, serves as a costimulator of T-cell function (Dinarello 2009). IL-10 activates mast cells and enhances the function of CD8^+^ T cells, NK cells, and B cells (Moore et al. 2001; Saraiva and O'Garra 2010). TGF-β promotes the production of peripheral (p)Treg, Th17, Th9, and Tfh cells and plays a pivotal role in the development and maturation of immune cells, as well as in regulating the immune response to pathogens (Lodyga and Hinz 2020; Sanjabi et al. 2017). These results provide evidence that the interaction between *P. multocida* OMVs and macrophages may lead to the presentation of associated antigens on the cell surface, activating the protective immune response of immune cells and mediating and promoting inflammation.

In summary, *P. multocida* OMVs can modulate cellular responses *in vitro*, augment phagocytic activity, promote proliferation and internalization, and induce cytokine secretion, thereby promoting the expedited clearance of pathogens. However, further investigations are needed to comprehensively elucidate the intricate regulatory mechanism of immune cells mediated by OMVs. This study provides the molecular and cellular basis for considering *P. multocida* OMVs as promising subunit vaccine antigen candidates, thereby bolstering the development of novel vaccines against *P. multocida* infections.

## Supplementary information

ESM 1 Table S1 List of the functions of the identified important *P. multocida* OMV proteins are described in order of decreasing PSMs. (XLSX 13 kb)

## Data Availability

The MS proteomics data have been deposited to the ProteomeXchange Consortium (http://proteomecentral.proteomexchange.org) via the PRIDE partner repository with the dataset identifier PXD046592.
